# Lysosomal Exocytosis of Olivacine on the Way to Explain Drug Resistance in Cancer Cells

**DOI:** 10.3390/ijms23116119

**Published:** 2022-05-30

**Authors:** Benita Wiatrak, Tomasz Gębarowski, Eddie Czwojdziński, Kazimierz Gąsiorowski, Beata Tylińska

**Affiliations:** 1 Department of Pharmacology, Faculty of Medicine, Wroclaw Medical University, Mikulicza-Radeckiego 2, 50-345 Wroclaw, Poland; benita.wiatrak@umw.edu.pl; 2 Department of Basic Medical Sciences, Wroclaw Medical University, 50-556 Wroclaw, Poland; 3 Department of Biostructure and Animal Physiology, The Wroclaw University of Environmental and Life Sciences, Kożuchowska 1/3, 51-631 Wroclaw, Poland; 4 Department of Toxicology, Wroclaw Medical University, 50-556 Wroclaw, Poland; eddie.czwojdzinski@umw.edu.pl; 5 Department of Organic Chemistry, Wroclaw Medical University, 50-556 Wroclaw, Poland; beata.tylinska@umw.edu.pl

**Keywords:** proton pump inhibitor, olivacine, multidrug resistant

## Abstract

Ellipticine is an indole alkaloid with proven antitumor activity against various tumors in vitro and a diverse mechanism of action, which includes topoisomerase II inhibition, intercalation, and cell cycle impact. Olivacine—ellipticine’s isomer—shows similar properties. The objectives of this work were as follows: (a) to find a new path of olivacine synthesis, (b) to study the cytotoxic properties of olivacine and ellipticine in comparison to doxorubicin as well as their impact on the cell cycle, and (c) to investigate the cellular pharmacokinetics of the tested compounds to understand drug resistance in cancer cells better. SRB and MTT assays were used to study the anticancer activity of olivacine and ellipticine in vitro. Both compounds showed a cytotoxic effect on various cell lines, most notably on the doxorubicin-resistant LoVo/DX model, with olivacine’s cytotoxicity approximately three times higher than doxorubicin. Olivacine proved to be less effective against cancer cells and less cytotoxic to normal cells than ellipticine. Olivacine proved to have fluorescent properties. Microscopic observation of cells treated with olivacine showed the difference in sensitivity depending on the cell line, with A549 cells visibly affected by a much lower concentration of olivacine than normal NHDF cells. An increased percentage of cells in G0/G1 was observed after treatment with olivacine and ellipticine, suggesting an impact on cell cycle progression, potentially via higher p53 protein expression, which blocks the transition from G0/G1 to the S phase. Ellipticine induced apoptosis at a concentration as low as 1 μM. It has been proved that the tested compounds (ellipticine and olivacine) undergo lysosomal exocytosis. Reducing exocytosis is possible through the use of compounds that inhibit the activity of the proton pump. Olivacine and ellipticine exhibited diverse cytotoxicity against a panel of cancer cells. Analysis of the lysosomal exocytosis of olivacine and ellipticine shows the need to look for derivatives with comparable anticancer activity but reduced weak base character.

## 1. Introduction

Exocytosis is the process of active transport of metabolites and particles out of cells into the extracellular space. In exocytosis, lysosomes (lysosomal exocytosis) can participate, and organelles are found in all cells except erythrocytes. The Golgi apparatus produce them. Lysosomes are 0.1–1.0 µm diameter vesicles surrounded by a single lipid–protein membrane. Inside these organelles, an acidic environment is required for the activity of many acid hydrolases. These hydrolytic enzymes break down various molecules, proteins, nucleic acids, fats, and carbohydrates delivered to the cell from the extracellular environment due to endocytosis or phagocytosis, as well as from intracellular damaged organelles (autophagy) [1].

In the process of exocytosis, lysosomes attach to microtubules and, on their surface, are transposed to the plasma membrane. Then, lysosomes connect to the plasma membrane in a process dependent on Ca^2+^ and release their content into the extracellular space. Lysosomal exocytosis takes part in physiological processes, including repairing the plasma membrane, endocytosis of large particles by macrophages, and degradation and regeneration of nerve cells. It is also suggested that lysosomal exocytosis is involved in removing anticancer drugs, which may contribute to drug resistance in cancer cells. Moreover, overexpression of lysosomal enzymes (cathepsins B, D, K, and L) may play a role in tumor invasion, metastasis, and progression. On the other hand, the leakage of cathepsin D into the cytosol may induce cell apoptosis [2].

There are many molecular targets in the treatment of cancer. Olivacine and its derivatives focus on three targets: interaction with deoxyribonucleic acid (DNA) topoisomerases, microtubule apparatus, and the effects on various enzymes responsible for stopping the cell cycle [3,4].

One of the compounds known for its anticancer activity (discovered in the 1960s) is ellipticine. It occurs naturally in several trees of the genera *Ochrosia*, *Rauvolfia*, *Aspidosperma*, and *Apocynaceae* [5]. Various mechanisms of the action of ellipticine and its derivatives have been described, e.g., intercalation, topoisomerase II inhibition, and impact on the cell cycle, including interactions with the p53 protein, Akt kinase, and c-Kit kinase [3,6,7,8]. Despite the antitumor properties of ellipticine, it has low bioavailability and solubility, which limits its usage [9].

Olivacine (1,5-dimethyl-6H-pyrido[4,3-b]carbazole) is a natural alkaloid, an isomer of ellipticine, isolated for the first time in 1958 from the bark of *Aspidosperma olivaceum* Müll Arg., an evergreen tree native to Brazil [10,11]. Alkaloids present in plants of the genus *Aspidosperma* exhibit antipyretic, analgesic, and antibacterial properties. *Aspidosperma* bark extracts are used in traditional medicine to treat malaria [10]. Due to the structural similarity of olivacine and ellipticine, it can be assumed that they should have similar properties.

Various methods have been described for the synthesis of olivacine, as well as its derivatives, some of which have shown promising anticancer properties. S16020, the best known of the olivacine derivatives, entered clinical trials where it was compared to methotrexate. The use of S16020 in radiotherapy has also been demonstrated, proving that using an intercalation factor and a topoisomerase II inhibitor may increase the effectiveness of radiotherapy [12,13].

Problems with using ellipticine, olivacine, and their derivatives are weak base character and hydrophobicity. After entering the interior of lysosomes, they are protonated in an acidic environment, resulting in that they cannot recross the lysosomal membrane. It is assumed that drug resistance results from the uptake of chemotherapeutic agents by lysosomes. Zhitomirsky and Assaraf showed a correlation between the number of lysosomes accumulating drug and cellular resistance to chemotherapeutic agents, including doxorubicin [2]. Doxorubicin is a hydrophobic anticancer drug used in the treatment of, among others, neuroblastoma, thyroid, breast, ovarian, and small cell lung cancers, Hodgkin’s disease, and acute lymphoblastic leukemia. The wide spectrum of doxorubicin applications raises controversies regarding the risk of developing multidrug resistance, probably due to lysosomal exocytosis.

In this study, an attempt was made to synthesize olivacine and then assess its biological activity in various cell lines. The results were compared with its isomer—ellipticine’s properties and the commonly used anticancer drug—doxorubicin. An important part of the study was the evaluation of olivacine exocytosis over 24 h on the A549 cell line.

## 2. Results and Discussion

### 2.1. Synthesis of Olivacine

**Figure 1:**
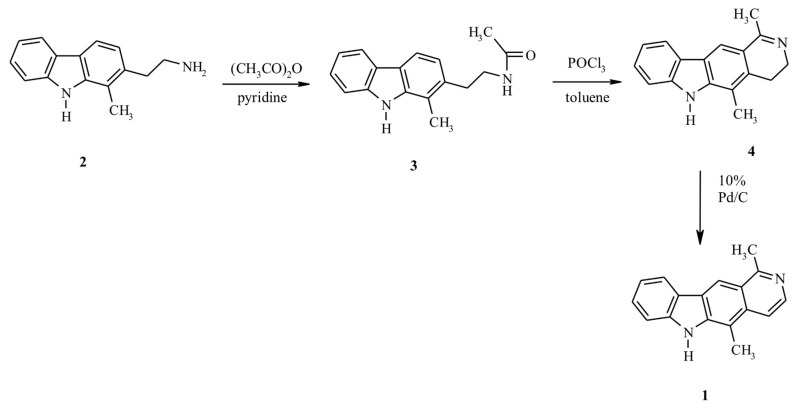
Synthesis of olivacine.

Olivacine **1** was synthesized according to Figure 1. The starting compound 2-(2-aminoethyl)-1-methyl-9*H*-carbazole **2** was prepared according to a previously described procedure [14,15]. Compound **3** was obtained by heating amine **2** in the mixture of acetic anhydride-pyridine (1:1). The cyclization of the resulting amid **3** with phosphorus oxychloride in boiling toluene gave derivative **4**. Finally, compound **4** was aromatized by dehydrogenation over 10% palladium on activated charcoal in boiling diphenyl ether to obtain olivacine **1** (mp. 320–325 °C) [16].

All melting points were determined on an Electrothermal Melting Point Apparatus Model 9100 and were uncorrected. H^1^ NMR spectra were recorded on a Bruker 300 at 300.14 MHz (Bruker, Rheinstetten, Germany) using TMS as the internal standard. In addition, column chromatography was carried out on silica gel (Merck Kieselgel 100; Merck, Darmstadt, Germany). All of the newly obtained compounds were analyzed for C, H, and N, and the analytical results were within ±0.4% of the theoretical values.

2-[2-(N-acetylamino)ethyl]-1-methyl-9*H*-carbazole 3

The 2-(2-aminoethyl)-1-methyl-9*H*-carbazole **2** (1.5 g, 6.7 mmol) was stirred for 2 h in the mixture of acetic anhydride-pyridine (1:1) 44 mL at room temperature. Evaporation to dryness under reduced pressure left a residue that was taken up to water (100 mL). After extraction with chloroform, the compound was crystallized from ethanol. Yield: 45%; mp. 161–162 °C. Anal. calc. for C_17_H_18_N_2_O: C, 76.66; H, 6.81; N, 10.52. Found: C, 76.57; H, 6.72; N, 10.61. ^1^H-NMR (DMSO-d_6_) δ: 1.79 (s, 3H, CO*CH*_3_), 2.50 (s, 3H, 1-CH_3_), 2.85 (t, 2H, α-CH_2_), 3.22 (m, 2H, β-CH_2_), 6.96 (d, 1H, J_3-4_ = 7.9 Hz, 3-H), 7.10 (m, 1H, 7-H), 7.32 (m, 1H, 6-H), 7.46 (d, J_8-7_ = 8.0 Hz, 1H, 8-H), 7.83 (d, J_4-3_ = 7.9 Hz, 1H, 4-H), 8.01 (m, 2H, 5-H, -NHCO), 11.02 (s, 1H, 9-NH).

1,5-dimethyl-3,4-dihydro-6H-pyrido[4,3-b]carbazole 4

The amide **3** (0.6 g, 2.2 mmol) was dissolved in boiling toluene (100 mL) and then treated dropwise with phosphorous oxychloride (10 mL). The reflux was continued for 1 h evaporation under reduced pressure afforded a residue that was taken up in water (10 mL), alkalis to pH 9–10 with sodium hydrogen carbonate and extracted with methylene chloride. The extract was dried over magnesium sulfate. After evaporation of the solvent, the solid was crystallized from methanol. Yield: 78%; mp.: 300–303 °C. Anal. calc. for C_17_H_16_N_2_: C, 82.22; H 6.49; N 11.28. Found: C, 82.11; H 6.38; N 11.20. ^1^H-NMR (DMSO-d_6_) δ: 2.81 (s, 3H, 5-CH_3_), 3.02 (s, 3H, 1-CH_3_), 3.15 (m, 2H, 3-CH_2_), 3.78 (m, 2H, 4-CH_2_), 7.28 (t, 1H, 9-H), 7.47 (t, 1H, 8-H), 7.60 (d, J_7-8_ =8.0 Hz, 1H, 7-H), 8.28 (d, J_10-9_ =7.7 Hz, 1H, 10-H), 8.80 (s, 1H, 11-H), 12.10 (s, 1H, 6-NH).

1,5-dimethyl-6H-pyrido[4,3-b]carbazole, Olivacine 1

The compound **4** (0.5 g, 2 mmol) was refluxed in diphenyl ether (30 mL) in the presence of 10% palladium on charcoal (0.10 g) for 30 min. The catalyst was filtered off, and the filtrate was cooled and diluted with 50 mL of hexane. The resulting residue was collected, washed with hexane, purified by chromatography on a silica gel column, and eluted with methylene chloride. Yield: 55%; mp.: 320–325 °C. Anal. calc. for C_17_H_14_N_2_: C, 82.90; H, 5.73; N, 11.37. Found: C, 82.75; H, 5.80; N, 11.29. ^1^H-NMR (DMSO-d_6_) δ: 2.52 (s, 3H, 5-CH_3_), 2.88 (s, 3H, 1-CH_3_), 7.23 (m, 1H, 9-H), 7.52 (m, 2H, 7-H, 8-H), 7.78 (d, J_4-3_ = 6.1 Hz, 1H, 4-H), 8.24 (d, J_3-4_ = 6.1 Hz, 1H, 3-H), 8.36 (d, J_10-9_ = 7.7 Hz, 1H, 10-H), 8.91 (s, 1-H, 11H), 11.33 (s, 1H, 6-NH).

### 2.2. Biological Activity

#### Inhibition of Cell Proliferation (Total Protein Content)

Tested compounds inhibited proliferation in all cancer cell lines (Table 1). Growth inhibition was calculated after olivacine, ellipticine, or doxorubicin administration to the culture. It was expected that a 50% decrease in total protein content (GI_50_) in cultures with olivacine would be observed at its low concentration. Olivacine was the most effective in MCF-7 cells (GI_50_ = 0.75 μM). Similar results were obtained in the case of CCRF/CEM (GI_50_ = 0.81 μM) and LoVo/DX cells (GI_50_ = 0.82 μM). An important observation is that olivacine was more effective against LoVo and LoVo/DX cells than doxorubicin. The effect of olivacine on LoVo/DX cells was slightly weaker than ellipticine. In the case of NHDF cells, olivacine was definitely less cytotoxic than ellipticine.

The MTT assay has shown that olivacine has a cytotoxic effect (according to the measurement of mitochondrial activity) depending on its concentration and the cell line used. The IC_50_ value for olivacine ranged from 12 to 26 μM and was different than for ellipticine. The olivacine activity was significantly weaker in all cell lines (Table 2) and most affected the CCRF/CEM and LoVo cells. As in the SRB assay, olivacine was also effective against LoVo/DX cells. Compared with doxorubicin, the cytotoxic effect of olivacine on the LoVo/DX cell line was approximately three times higher. Olivacine was slightly more cytotoxic than doxorubicin in the normal cell line (NHDF).

Our research has proven that ellipticine, a better known olivacine isomer, may have cytostatic effects even at low concentrations (1–4 μM). Stiborová et al. provided information on the activity of ellipticine, showing that after 48 h exposure, IC_50_ values for MCF-7 and CCRF/CEM cells were 1.25 ± 0.13 μM and 4.70 ± 0.48 μM, respectively [17]. Kuo had similar results for MCF-7 cells, in which IC_50_ was 1.52 μM [18]. However, the results obtained in the MTT assay in our study differ (Table 2), suggesting lower ellipticine activity in MCF-7 cells (IC_50_ = 4.67 ± 0.54 μM). The calculated IC_50_ value for CCRF/CEM cells was comparable with that obtained by Stiborová et al. (IC_50_ = 5.86 ± 1.44 μM). Our results also show that olivacine is less cytotoxic for normal cells (NHDF) than ellipticine and is similarly as cytotoxic as doxorubicin.

### 2.3. Rate of Apoptosis and Evaluation of the Cell Cycle

**Figure 2:**
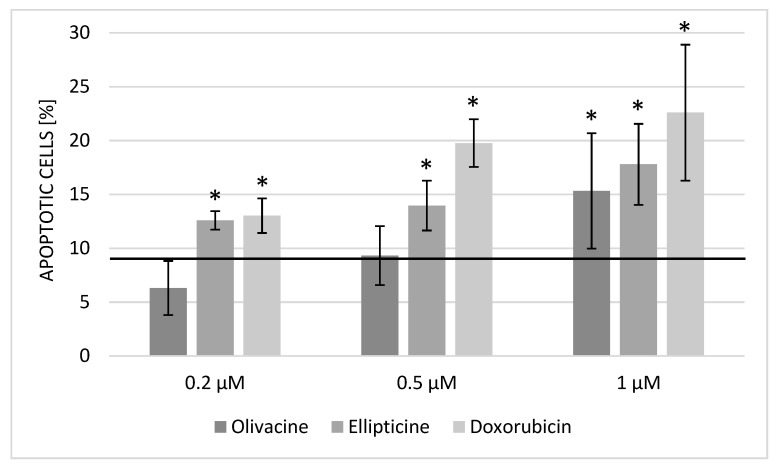
Rate of apoptosis after 24 h incubation with olivacine, ellipticine, or doxorubicin. Apoptotic cells in the control culture: 8.59 ± 0.97%. Statistical significance calculated with *t*-test (* *p* < 0.05).

Apoptosis is a strictly regulated mechanism of cell death. In cancer diseases, this process is often disturbed. One of the main features of cancer cells is cell cycle dysregulation, which increases the number of cells in the cell division phase. The results showed that olivacine induced apoptosis at a concentration of 1 μM, although its effect was slightly weaker than ellipticine (Figure 2).

The effect of most cytostatic drugs is related to the cell cycle [19]. These compounds interfere with the DNA structure, affecting its synthesis and increasing the expression of many genes, leading to cell cycle arrest [20]. Previous studies have shown that ellipticine is a potent cell cycle inhibitor.

The cell division inhibition effect was observed using 1 µM. No cell cycle inhibition was observed in the cell culture treated with olivacine at the lower concentrations tested such as 0.2 µM and 0.5 µM. In cells treated with olivacine or ellipticine, an increased percentage of cells in the G_0_/G_1_ phase was observed, suggesting that these compounds may stop cell cycle progression in the G_0_/G_1_ phase (Table 3). The p53 protein also affects cell cycle regulation. As p53 increases, higher p21 protein expression blocks the transition from G_0_/G_1_ to the S phase. The p53 increases the number of apoptotic cells and stops the cell cycle, affecting the activation of DNA damage repair pathways. On this basis, it can be assumed that ellipticine and olivacine increase the expression of p53 protein. Similar results were obtained by Kuo for MCF-7 cells [18] and Kim for RL95-2 cells [21].

On the other hand, Tao et al., based on the T24 cell study, argued that ellipticine stops the cell cycle in the G_2_/M phase instead of G_0_/G_1_ [22]. Mizumoto, studying the effect of ellipticine on pancreatic cancer cells, showed that low concentrations (1 μM) of ellipticine could arrest cell cycle progression in both G_1_ and G_2_/M phases [23]. Differences in results may suggest a certain level of ellipticine specificity for some cell lines. Russel et al. reported that the effect of the ellipticine on the cell cycle may also depend on its concentration—for MV4–11 cells at 5 μM concentration caused the arrest of the cell cycle at the G_2_/M checkpoint. In contrast, higher concentrations resulted in an arrest in the G_1_ phase [24].

Ohashi et al. were the first to suggest that even short exposure to ellipticine can restore the function of p53 protein. They observed that ellipticine induced apoptosis at 8 and 10 μM concentrations in Saos-2 cells [25]. Our results suggest that ellipticine can induce apoptosis in CCRF/CEM cells at a much lower concentration (1 μM). Furthermore, Savorani et al.’s research shows that treatment of CCRF/CEM cells with ellipticine may stop the cell cycle in the G_0_/G_1_ phase [26]. These observations are consistent with our results—after exposure to olivacine or ellipticine, we observed blockade of the cell cycle in the G_0_/G_1_ phase in CCRF/CEM cells.

### 2.4. Cell Morphology

After 24 h incubation with olivacine, A549 and NHDF cells were observed under an EVOS microscope with a fluorescence filter. Microscopic observation confirmed that olivacine exhibits natural green fluorescence similar to ellipticine. Based on these observations, a visual assessment of morphology was performed.

**Figure 3:**
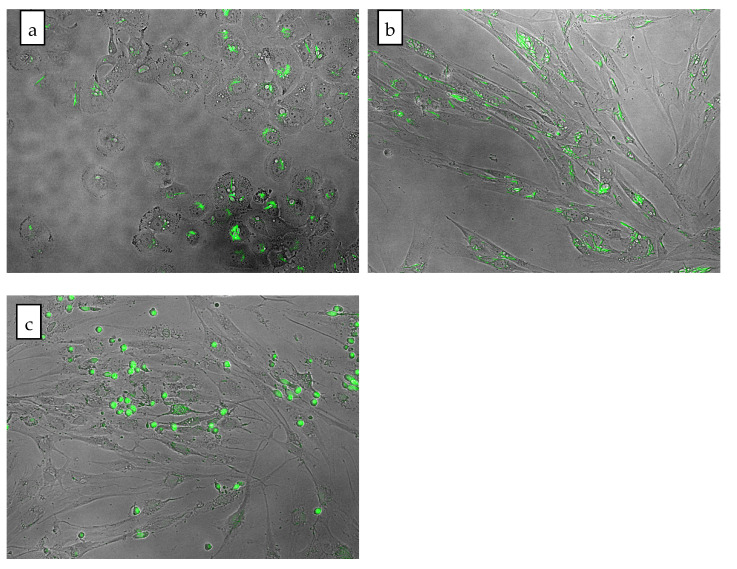
Comparison of cell morphology after 24 h incubation with olivacine: (**a**) A549 cell line, 2 µM olivacine; (**b**) NHDF cell line, 5 µM olivacine; (**c**) NHDF cell line, 20 µM olivacine.

Cytosol degradation was observed after adding 2 μM olivacine to the A549 cell culture (Figure 3a). On the other hand, the same concentration added to normal cells (NHDF) did not negatively affect cell morphology. Moreover, the morphology of NHDF cells was normal even after 24 h incubation with 5 μM olivacine (Figure 3b). We also compared the morphology of A549 and NHDF cells after treatment with 20 μM olivacine. The effect on the morphology of both cultures was noticeable but varied depending on the cell line. The cells of the tumor line have been completely degraded, while only a few fibroblast cells have been disintegrated in the field of view.

Microscopic observations have shown that the effect of olivacine depends on both the cell line and the concentration. For example, A549 cells were more sensitive to olivacine, with growth inhibition visible at 2 μM, while NHDF cells grew even at a 20 μM concentration of olivacine (Figure 3c).

### 2.5. Exocytosis of Olivacine

**Figure 4:**
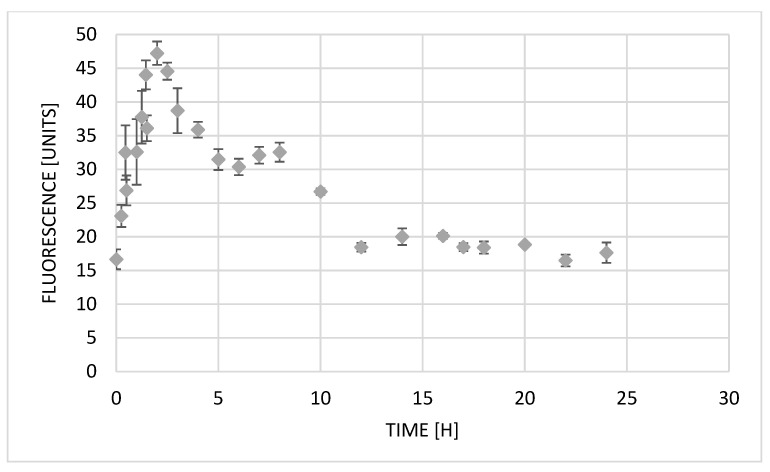
Changes in fluorescence intensity in A549 cells after treatment with olivacine (10 µM).

The A549 line, which was also used in this study, is often used to assess exocytosis in in vitro studies [27,28]. Based on automatically taken images under a Juli microscope every 15 min, we analyzed the change in olivacine (10 µM) fluorescence in cells over time (Figure 4). The results allowed us to evaluate the cellular pharmacokinetics of olivacine.

Immediately after the administration of olivacine, a rapid increase in fluorescence is observed for more than 2 h, which indicates an increase in the substance contained inside the cells. The maximum value of fluorescence intensity, which also means the highest concentration of olivacine in the cells, was reached after 2 h 15 min. After uptake of olivacine into the cell, it can be transferred to the nucleus or lysosomes. In the second case, it can cause lysosomal stress. The subsequent decrease in fluorescence may suggest the occurrence of the lysosomal exocytosis process [2]. With an increase in the amount of compound in the lysosome, calcium ions (Ca^2+^) are released into the cytoplasm. Ca^2+^ activate calcineurin, which dephosphorylates transcription factor EB (TFEB). Therefore, TFEB is translocated into the cell nucleus, activating CLEAR (coordinated lysosomal expression and regulation) genes [1]. Activated signaling pathways cause lysosomes to bind to microtubules and move toward the cell membrane. Then, in the presence of Ca^2+^, lysosomes attach to the cell membrane, releasing their content into the extracellular space. In our work, the decrease in fluorescence intensity 2 h 15 min after olivacine administration is probably the result of the described lysosomal exocytosis process. Zhitomirsky and Assaraf have shown that this process may increase cellular resistance to the cytotoxic effects of chemotherapeutic drugs. It is also suspected that some lysosomal enzymes released due to the drug-induced lysosomal endocytosis may be involved in tumor invasion, metastasis, and progression.

**Figure 5:**
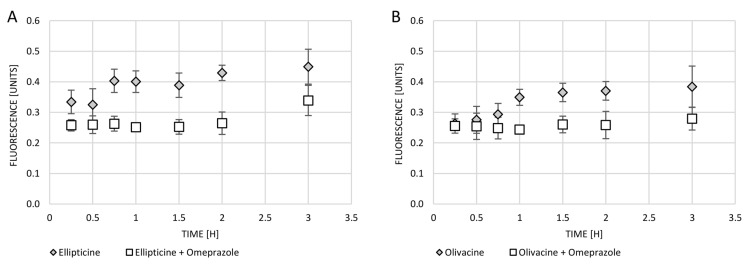
Changes in fluorescence intensity in the supernatant collected from A549 cells over time after treatment with: (**A**) ellipticine (10 µM) or ellipticine (10 µM) and omeprazole (200 µM); (**B**) olivacine (10 µM) or olivacine (10 µM) and omeprazole (200 µM).

Omeprazole is a proton pump inhibitor that prevents cell acidity. The 200 µM omeprazole was added to the cell culture for 1 h to see whether this would avoid the exocytosis of test compounds (olivacine and ellipticine). After incubation with omeprazole, the cells were washed and then treated with olivacine or ellipticine. Blocking of the proton pump with omeprazole has been observed to protect against exocytosis of both olivacine and ellipticine. As shown in the graph, the fluorescence of neither olivacine nor ellipticine decreases over time (Figure 5).

The fluorescence of tested compounds in cells was checked. When the cells were only treated with olivacine or ellipticine, increasing fluorescence in the medium with the passage of incubation time was observed. Earlier use of omeprazole affects the blockage of the proton pump, which inhibits exocytosis of the tested compounds. One hour incubation of cells with omeprazole affected the blockage of exocytosis of olivacine for at least 3 h, while exocytosis of ellipticine was only observed after 3 h.

**Figure 6:**
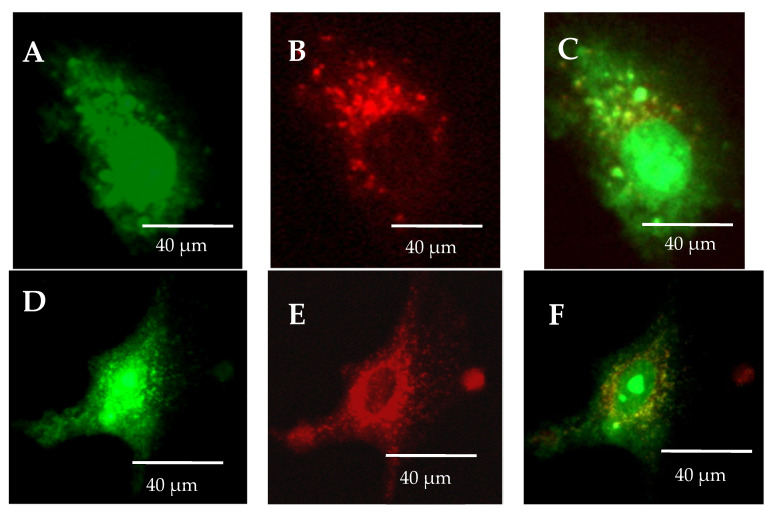
Differences in the occurrence of olivacine accumulation depending on the administration: (**A**–**C**) administration of olivacine alone causes the accumulation of olivacine (green) in lysosomes (red); (**D**–**F**) administration of olivacine and omeprazole causes olivacine accumulates in the cytoplasm and not in the lysosomes.

**Figure 7:**
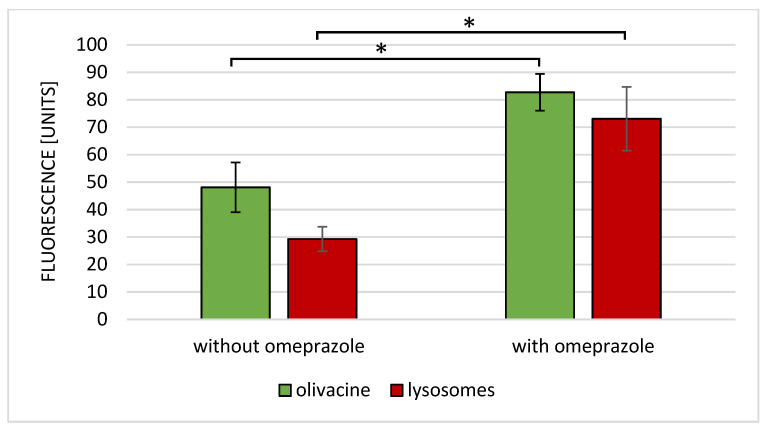
Mean fluorescence of olivacine and lysosomal absorbance after administration of olivacine with or without omeprazole. * *p* < 0.05.

The lack of omeprazole causes the accumulation of olivacine in the lysosomes, as shown in Figure 6A. Olivacine is then removed from the cells by the lysosomes. On the other hand, the administration of omeprazole and olivacine resulted in the formation of olivacine in the cytoplasm and not in the lysosomes (Figure 6B). Coadministration of omeprazole and olivacine increases the accumulation of olivacine in the nucleus (Figure 6D,F). From Figure 7, it should be noted that the increase in mean intracellular fluorescence is statistically significantly greater when olivacine and omeprazole are administered simultaneously.

## 3. Conclusions

This article presents results suggesting the anticancer activity of ellipticine and olivacine on various cell lines. The study confirmed that ellipticine is cytotoxic to cancer cells, can inhibit the cell cycle, and induce apoptosis. Olivacine has a similar, though smaller, effect on cancer cells than ellipticine but is also less toxic to normal cells. Both ellipticine and olivacine show significantly more potent activity in drug-resistant cells (LoVo/DX) than doxorubicin.

Lysosomal exocytosis applies to ellipticine and olivacine. For this reason, it is planned to search for their derivatives showing similar antitumor activity but with a reduced problem of exocytosis. Furthermore, the process of olivacine lysosomal exocytosis can be inhibited by simultaneous administration of a proton pump inhibitor.

The obtained results will allow for better analysis and interpretation of the biological activity of olivacine and its derivatives. Modifications of the compound (new derivatives) are carried out to improve its activity and limit its removal from the cell.

## 4. Materials and Methods

### 4.1. Preparation of Compounds for Biological Assays

Tested compounds (olivacine, ellipticine, doxorubicin) were dissolved in DMSO and later in a culture medium to obtain the desired concentration (0.2–20 μM). The final DMSO concentration did not exceed 0.2%.

### 4.2. Cell Lines

The anticancer activity of tested compounds was assessed on five human cell lines selected from the panel of tumor cell lines recommended by the National Cancer Institute: A495 (lung carcinoma), CCRF/CEM (acute lymphoblastic leukemia), LoVo and LoVo/DX (two types of colorectal: non-resistant and resistant to doxorubicin), and MCF-7 (breast adenocarcinoma). In addition, the NHDF cell line (normal human dermal fibroblasts) was used to estimate cytotoxicity to noncancer cells. The A549, CCRF/CEM, LoVo, LoVo/DX, and MCF-7 cells were obtained from ECACC, and NHDF cells from LONZA, Belgium. The A549 and MCF-7 cells were grown in EMEM medium, CCRF/CEM cells in RPMI-1640, LoVo and LoVo/DX cells in DMEM/F-12, and NHDF cells in DMEM without phenol red. The cells were seeded in 7.5 × 105 cells per well in the biological assay, as recommended by the National Cancer Institute [29,30]. All culture media were supplemented with L-glutamine, penicillin, streptomycin, and 10% fetal bovine serum (FBS). Cells were incubated at 37 °C and 5% CO_2_.

### 4.3. Inhibition of Cell Proliferation (Total Protein Content)

One of the most popular in vitro tests to evaluate the activity of compounds with potential antitumor activity is the SRB assay [30]. The sulforhodamine B dye is used, which binds to the protein at the appropriate pH. The assay allows for assessing cell mass and, indirectly, their proliferation. The advantage of the assay is that it measures cellular protein content and not the activity of any enzymes [31]. The SRB assay was carried out according to the National Cancer Institute procedure. Cells were incubated in a culture medium for 24 h in 96-well plates. After 24 h, the control plate for each cell line was fixed with TCA to evaluate cell culture before treatment with tested compounds. The compounds were added to the other plates (at five concentrations of 1–20 μM) and incubated for another 48 h. Cell cultures were then fixed by adding 50% TCA at 4 °C for 1 h. After removing the supernatant, the plates were washed with water and dried. Cells were stained with 0.4% sulforhodamine B solution in 1% acetic acid for 10 min at RT. The unbound dye was removed by washing with 1% acetic acid. After drying, the dye attached to the proteins was dissolved in a 10 mM Trizma base. The absorbance was measured at 490 nm using a Victor2 microplate reader (PerkinElmer, Waltham, MA, USA). Growth inhibition was calculated following the NCI guidelines and expressed as the GI_50_ ratio, i.e., the concentration of the compound in which the amount of protein was reduced by half compared with the control.

### 4.4. Cytotoxicity (Mitochondrial Activity)

An assessment of the mitochondrial cell activity after applying tested compounds was carried out using the test MTT assay, recommended in the ISO 10993-5:2009 standard. As in the SRB assay, the cells were exposed to tested compounds after 24 h of initial incubation in 96-well plates. The MTT solution (1 mg/mL) was added to each well, and cells were incubated for 2 h. Finally, the supernatant was removed, and the formazan crystals were dissolved in isopropanol. After 30 min, absorbance at 570 nm in Victor2 microplate reader (PerkinElmer). Cell viability was determined as the IC_50_ ratio, i.e., the concentration of the compound in which the viability was reduced by half compared with the control.

### 4.5. Rate of Apoptosis

Apoptosis is a process of programmed cell death. After exposure to tested compounds, the apoptosis rate was measured using Tali Apoptosis Kit (Invitrogen, Thermo Fisher Scientific, Waltham, MA, USA). In the process of apoptosis, phosphatidylserine (PS) is transferred to the outer side of the cell membrane. The assay uses the ability of fluorophore-labeled annexin V to identify apoptotic cells by binding to exposed PS. The CCRF/CEM cells were incubated in RPMI-1640 medium in 24-well plates for 24 h (37 °C, 5% CO_2_). Then, tested compounds were added at three different concentrations (0.2, 0.5, and 1 μM) and incubated for 24 h. Negative control was the culture of cells not exposed to apoptosis-inducing factors. After incubation with tested compounds, the cells were harvested, centrifuged, and the supernatant removed. Cells were suspended in annexin V binding buffer (ABB), and Alexa Fluor 488 annexin V conjugate was added to each sample. The cells were then incubated for 20 min in the dark at RT, centrifuged, and resuspended in ABB with propidium iodide (PI). After 5 min incubation, the stained samples were transferred to Tali Cellular Analysis Slides and analyzed using an image-based cytometer Arthur (NanoEnTek Inc., Seoul, Korea).

### 4.6. Evaluation of the Cell Cycle

It is expected that tested compounds affect cell division processes (intense in cancer). Therefore, the effect of compounds on the cell cycle was assessed by quantifying the content of cellular DNA in CCRF/CEM cells with Tali Cell Cycle Kit (Invitrogen). Cells were prepared by 24 h initial incubation in RPMI-1640 medium followed by the addition of tested compounds for a further 24 h. Subsequently, cells were harvested, centrifuged, washed, dissolved in PBS, and centrifuged again. After removing PBS, the cells were resuspended in Tali Cell Cycle Solution and incubated for 20 min in the dark at RT. Finally, the cells were transferred to the Tali Cellular Analysis Slides and analyzed using an image-based cytometer Arthur (NanoEnTek Inc.).

### 4.7. Cell Morphology and Exocytosis of Olivacine

Olivacine, similar to ellipticine, is a compound with fluorescent properties. Therefore, by analyzing the intensity of fluorescence over time, it is possible to assess the cellular pharmacokinetics of the compounds. At the same time, cell morphology was also assessed.

The cells were seeded into a 96-well plate and cultured for 24 h (37 °C, 5% CO_2_) for cell adhesion to the surface and regeneration. The following day, olivacine or ellipticine was added for 1 h at a concentration of 10 µM. The culture plate was placed under a Juli microscope, and the device with the plate inside was put in the CO_2_ incubator. Pictures were taken every 15 min for 24 h. We have prepared software that allowed us to measure the fluorescence intensity of the indicated cells. Fluorescence analysis was performed for all images (every 15 min) during a period of rapid change in fluorescence intensity and less often at a later period when it stabilized. Three independent experiments were carried out, always analyzing 50 selected cells.

When the cells were incubated with the test compound, the supernatant took 2 μM every 15 min into another microplate, and fluorescence was measured using a Varioskan LUX plate reader (Thermo Fisher Scientific) to assess whether the concentration of test compounds increased in the medium.

### 4.8. Evaluation of Proton Pump Activity

To assess whether the exocytosis of olivacine and ellipticine from cancer cells is lysosomal, a proton pump blocking test was performed. Omeprazole was added into A549 cells at a concentration of 200 µM for 1 h. The cell culture was then washed twice with PBS, and the cells were treated with tested compounds. The cell culture was assessed using an Evos FL microscope. The lysosomes were stained using LysoTracker™ Deep Red kit (L12492; Invitrogen). Cultured cells were evaluated using an Evos FL microscope, taking photos every 15 min. After treatment with olivacine or olivacine and omeprazole, analysis of mean cell fluorescence was performed from five independent replicates. In addition, 14 pictures were taken from each replicate, and 100 cells were analyzed by photomicrography using the ImageJ platform.

### 4.9. Statistical Analysis

Assays were carried out in five independent experiments in triplicate. All results were analyzed using the *t*-test. Results of SRB and MTT assays are presented as the GI50 and IC50 ratios, respectively—concentrations at which 50% growth/viability inhibition should be observed. The number of apoptotic cells is shown as a mean and standard deviation. The evaluation results of the cell cycle were expressed as the percentage of cells in a given phase.

## Figures and Tables

**Table 1:** SRB assay results (GI_50_ values) after treatment with olivacine or ellipticine in A549, MCF-7, LoVo, LoVo/DX, CCRF/CEM, and NHDF cells.

Cell Line	GI50 [µM]			Significance of the Difference Between Olivacine and Ellipticine
	Olivacine	Ellipticine	Doxorubicin	
A549	0.93 ± 0.06	0.72 ± 0.06	1.92 ± 0.35	*p* < 0.05
MCF-7	0.75 ± 0.07	1.25 ± 0.77	1.44 ± 0.23	*p* > 0.05
LoVo	1.02 ± 0.21	0.95 ± 0.25	1.62 ± 0.45	*p* > 0.05
LoVo/DX	0.82 ± 0.08	0.75 ± 0.22	14.09 ± 3.42	*p* > 0.05
CCRF/CEM	0.81 ± 0.14	0.77 ± 0.17	0.74 ± 0.02	*p* > 0.05
NHDF	14.77 ± 3.77	4.44 ± 0.74	13.65 ± 5.29	*p* > 0.05

Cytotoxicity (mitochondrial activity).

**Table 2:** MTT assay results (IC_50_ values) after treatment with olivacine or ellipticine in A549, MCF-7, LoVo, LoVo/DX, CCRF/CEM, and NHDF cells.

Cell Line	IC_50_ [µM]			Significance of the Difference Between Olivacine and Ellipticine
	Olivacine	Ellipticine	Doxorubicin	
A549	17.84 ± 7.82	12.92 ± 2.36	13.75 ± 1.03	*p* < 0.05
MCF-7	18.50 ± 2.41	4.67 ± 0.53	4.33 ± 0.55	*p* < 0.05
LoVo	12.66 ± 1.08	3.27 ± 0.83	10.15 ± 1.27	*p* < 0.05
LoVo/DX	26.33 ± 4.64	15.05 ± 1.73	86.35 ± 25.42	*p* < 0.05
CCRF/CEM	10.97 ± 3.30	5.86 ± 0.51	1.03 ± 0.02	*p* < 0.05
NHDF	15.27 ± 1.95	8.98 ± 1.44	18.19 ± 4.46	*p* < 0.05

**Table 3:** Percentage of CCRF/CEM cells in various cell cycle phases after 24 h exposure to olivacine, ellipticine, and doxorubicin.

	Percentage of Cells in a Given Phase [%] (SD)		
	G_0_/G_1_	S	G_2_/M
Control	33.1 (2.4)	21.7 (2.2)	45.2 (4.9)
Olivacine	37.0 (1.9)	22.5 (2.3)	40.5 (0.9)
Ellipticine	36.9 (2.2)	22.8 (3.9)	40.3 (0.8)
Doxorubicin	27.9 (3.0)	34.7 (4.4)	37.4 (1.7)

## Data Availability

Not applicable.
